# Ten Years of Integrative Carbohydrate Structure Validation with *Privateer*

**DOI:** 10.1093/glycob/cwag068

**Published:** 2026-08-22

**Authors:** Lucy Schofield, Jordan Dialpuri, Haroldas Bagdonas, Lou Holland, Phuong Thao Pham, Shuai Wang, William Cook, Jon Agirre

**Affiliations:** 1 York Structural Biology Laboratory, Department of Chemistry, University of York, York YO10 5DD, UK; 2 MRC Laboratory of Molecular Biology, Francis Crick Avenue, Cambridge CB2 0QH, UK

**Keywords:** cryo-EM, crystallography, glycans, glycoproteins, validation

## Abstract

Understanding glycoprotein structures is critical for understanding their biological roles, yet despite the moderate size of the glycans, determining the structure of these atomic models presents unique challenges. Originally designed to help mine the Protein Data Bank for monosaccharides and their context, the *Privateer* carbohydrate validation software has evolved to become ubiquitous in carbohydrate model building workflows. *Privateer* detects issues affecting sugar stereochemistry, regiochemistry, pyranoside and furanoside ring conformation, as well as overall glycan conformation and composition. Supporting both X-ray crystallography and cryo-electron microscopy data, *Privateer* can communicate with refinement software to help restrain sugar rings to their minimal-energy conformations. In this work, we reflect on a decade of *Privateer*, documenting its core processes and best practices to serve as the primary citation for the software.

## Introduction

**Figure 1:**
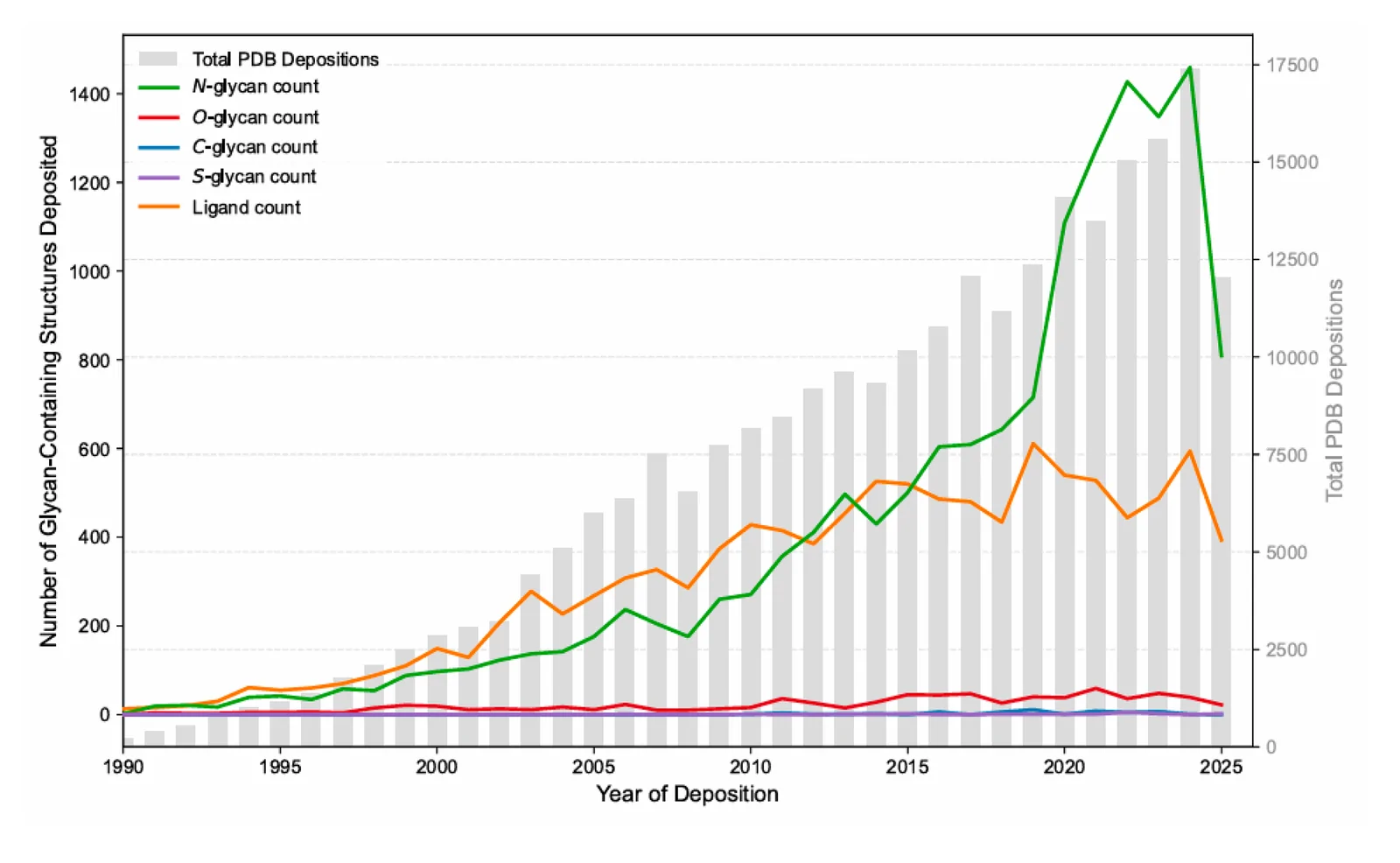
Distribution of carbohydrate-containing structure deposition in the Protein Data Bank over time. Grey bars (right axis) show the total number of structures deposited annually. Lines track structure deposition counts for specific carbohydrate categories (left axis): *N-*glycans, *O-*glycans, *C-*glycans, *S-*glycans and ligands. The analysis indicates around 10% of yearly deposits are sugar-containing structures. The apparent decline in deposited structures for 2025 likely reflects structures that are still undergoing deposition review or on hold awaiting publication. Data were extracted from the *Privateer Database* (Dialpuri, Bagdonas, Schofield, Pham, Holland, and Agirre 2024). Figure 1 alt text: Dual-axis chart showing the annual deposition of structures in the PDB over time, tracking total depositions alongside specific glycan categories.

Carbohydrates are one of the most structurally diverse classes of macromolecules, owing to their extensive branching patterns and stereochemical variation (Schachter et al. 1982; Werz et al. 2007). They play a central role in many molecular mechanisms, including cellular recognition, immune evasion, and viral pathogenesis (Bangaru et al. 2020; Lenza et al. 2023). Consequently, determining the structure of glycoproteins has long been important for understanding the molecular basis of disease and developing therapeutics, as illustrated by the design of the SARS-CoV-2 mRNA vaccines (Bangaru et al. 2020; Seeberger et al. 2022; Lenza et al. 2023). Structural studies of glycoproteins demonstrate that glycans are rarely passive post-translational modifications; instead, they actively influence protein folding and alter binding interfaces through protein-carbohydrate interactions (Shental-Bechor and Levy 2008; Cummings et al. 2022). Capturing these glycans is therefore essential for observing macromolecular structures in their native, functional states. In the Protein Data Bank (PDB), carbohydrate-containing structures consistently represent ~ 10% of the total annual depositions, making them the most common post-translational modification in the repository (see Figure 1) (Berman et al. 2000; Agirre 2017; Dialpuri, Bagdonas, Schofield, Pham, Holland, and Agirre 2024; Schofield et al. 2024).

Despite their biological importance and consistent deposition, the structural determination of sugars is often challenging. Their high flexibility, combined with microheterogeneity and complex branching networks, often results in difficult-to-interpret density in both X-ray crystallography and cryo-electron microscopy (cryo-EM) maps, particularly with low-resolution data (Agirre et al. 2017). Structural complexities, including anomeric stereochemistry, regiochemical branching, and ring conformations, make carbohydrate modelling uniquely difficult. As a result of this, the quality of these structures has historically lagged behind that of proteins and nucleic acids (Agirre, Davies, et al. 2015). This discrepancy was largely driven by a deficit in software that provided automatic pipelines to enforce correct sugar chemistry, as standard macromolecular building and refinement tools were optimised for proteins. Analysis of the PDB revealed widespread modelling errors, including structures containing high-energy ring-conformations, incorrect anomeric configurations, or biologically impossible linkages that violate fundamental stereochemical rules (Agirre, Davies, et al. 2015). In fact, a 2004 study identified nomenclature errors in approximately 30% of carbohydrate deposition (Lütteke et al. 2004). Further, a 2015 study found that 30% of deposited structures featured sugars modelled in high-energy conformations, which is surprising given that such distortion typically only occurs within enzyme active sites (Agirre, Davies, et al. 2015).

**Figure 2:**
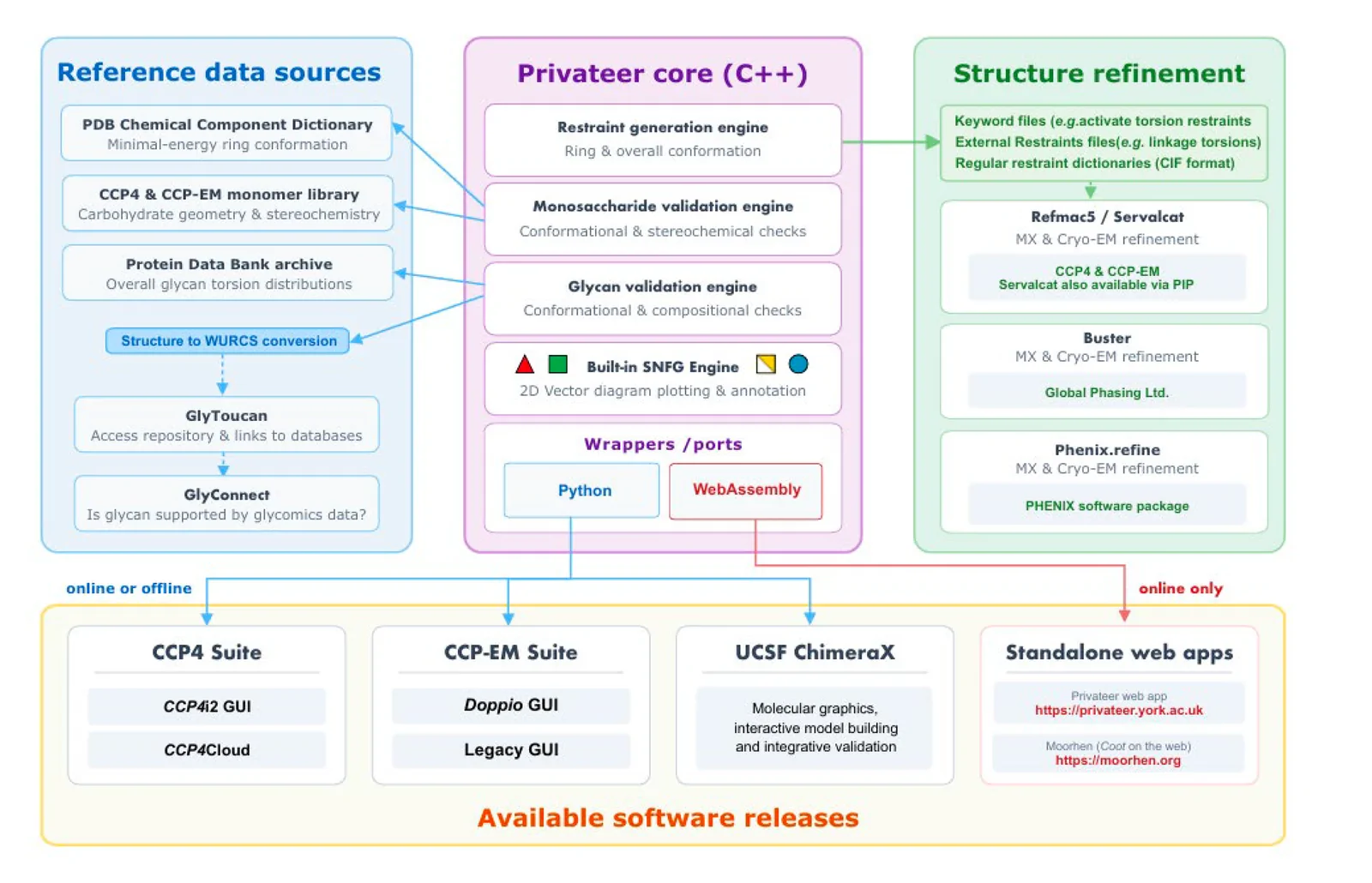
The architecture of *Privateer* and its links to other community resources. The core of *Privateer* is written in C++, which relies on a number of data sources, including the PDB Chemical Component Dictionary, CCP4 and CCP-EM Monomer Library, a PDB-derived database of linkage torsion angles, as well as offline mirrors of *GlyConnect* and *GlyToucan* (Westbrook et al. 2015; Tiemeyer et al. 2017; Alocci et al. 2019; Agirre et al. 2023). Restraints can be outputted for use in a number of refinement engines. *Privateer* is available via the CCP4 or CCP-EM Software Suites, as well as a plugin for *ChimeraX* (Agirre et al. 2023; Meng et al. 2023; Holland et al. 2026). It is also available in *Moorhen*, a *Coot*-based model building web application, as well as via the *Privateer* web app (Emsley et al. 2010; Debreczeni and Emsley 2012). Figure 2 alt text: Flowchart showing the architecture of *Privateer*, its data sources, and outputs.

To rectify some of these issues, *PDB-CARE* was developed to automatically check the connectivity and naming of oligosaccharides, while the *CARP* server allowed users to compare torsional data against existing structures (Lütteke and von der Lieth 2004; Lütteke et al. 2005). While valuable for correcting nomenclature and linkages, the field still lacked a unified approach capable of assessing the complex conformations that sugar rings can adopt, as well as evaluating how well a sugar fits the experimentally-derived density. Assessing the fit is key, as density serves as a three-dimensional map of atomic positions derived from experimental measurements into which the atomic model must be built and validated. Ten years ago, to address these systematic issues in the PDB, the software package *Privateer* was developed (Agirre, Iglesias-Fernández, et al. 2015). It has evolved from a conformational analysis program into a multi-functional carbohydrate structure validation tool that is easily integrated into structural biology pipelines. Today, it is widely accessible to the scientific community, available in both *CCP4i2* and *CCP4Cloud* of the CCP4 Software Suite, CCP-EM, as a plug-in for *ChimeraX*, via the *Privateer* web application (https://privateer.york.ac.uk), via *Moorhen* (https://moorhen.org/), as a stand-alone command line program, or a python API (Emsley et al. 2010; Agirre et al. 2023; Burnley et al. 2023; Meng et al. 2023; Dialpuri, Bagdonas, Schofield, Pham, Holland, Bond, et al. 2024; Holland et al. 2026). The main functionality of *Privateer* involves the detection and validation of carbohydrates by calculating Cremer-Pople puckering parameters (Cremer and Pople 1975), as well as validating a sugar’s fit-to-density by calculating real-space correlation coefficients (RSCC) in electron density maps, a standard metric used in crystallography to assess how well an atomic model fits the local density. *Privateer* can be used to validate *N*-, *O*-, *C*- and *S*-linked glycans across both linear and branched topologies. Complex polysaccharides such as glycosaminoglycans and glycolipids are also supported by *Privateer*. It can also be applied to protein-sugar complexes, including structures of antibodies, enzymes, lectins and receptors (Pinto et al. 2020; Akkermans et al. 2022; Morais et al. 2023; Trenker et al. 2024). Crucially, *Privateer* does not only flag errors, it also generates restraint dictionaries that can be fed directly back into refinement engines like *REFMAC5, Servalcat, BUSTER and PHENIX.refine*, enabling corrections of distorted rings during structure refinement (Murshudov et al. 2011; Afonine et al. 2012; Bricogne et al. 2017; Yamashita et al. 2021; Atanasova et al. 2022; Yamashita et al. 2023). An overview of *Privateer’s* main architecture and functionality can be seen in Figure 2.

Going forward, as structural biology moves towards automation and machine-learning driven structure solution, the structural validation of carbohydrate structures deposited into the PDB is extremely important. As structures in the PDB are continually used to inform drug design and train machine learning algorithms, carbohydrate validation is essential to prevent misinterpretation of glycoprotein and protein-glycan complex structures. In this paper, we will reflect on a decade of *Privateer*, including its recent extensions, scientific impact, and ongoing role in advancing structural glycobiology.

## Algorithm details

### Cremer-Pople parameters flag high-energy conformations

One key aspect of carbohydrate chemistry is the principle that monosaccharides exist in a dynamic equilibrium between open-chain and cyclic structures, adopting six-membered pyranose or five-membered furanose rings. Unlike planar aromatic rings, carbohydrates exhibit ring puckering to minimise ring strain and steric clashes, by favouring staggered rather than eclipsed substituent orientations. Consequently, these rings have strong preferences for specific low-energy conformations. For pyranoses, these low-energy states are ^4^C_1_ chair conformation for d-pyranoses, and ^1^C_4_ for l-pyranoses. However, exceptions exist, notably sialic acid, a sugar with high biological importance due to its role in immune response and viral entry. It is an L-pyranose that adopts a ^2^C_5_ chair conformation due to its unique C2 anomeric numbering and extended carbon backbone. While genuine transitions into high-energy conformations are biologically relevant, they are rare and typically restricted to the transition states that occur in enzyme active sites. In the absence of this enzymatic stabilisation, the modelling of high-energy conformers such as envelopes (^3^E), boats (^2.5^B) and skew-boats (^1^S_5_) is likely a modelling or refinement error, with high-energy conformations fit to noisy or fragmented electron density (Agirre, Davies, et al. 2015).

**Figure 3:**
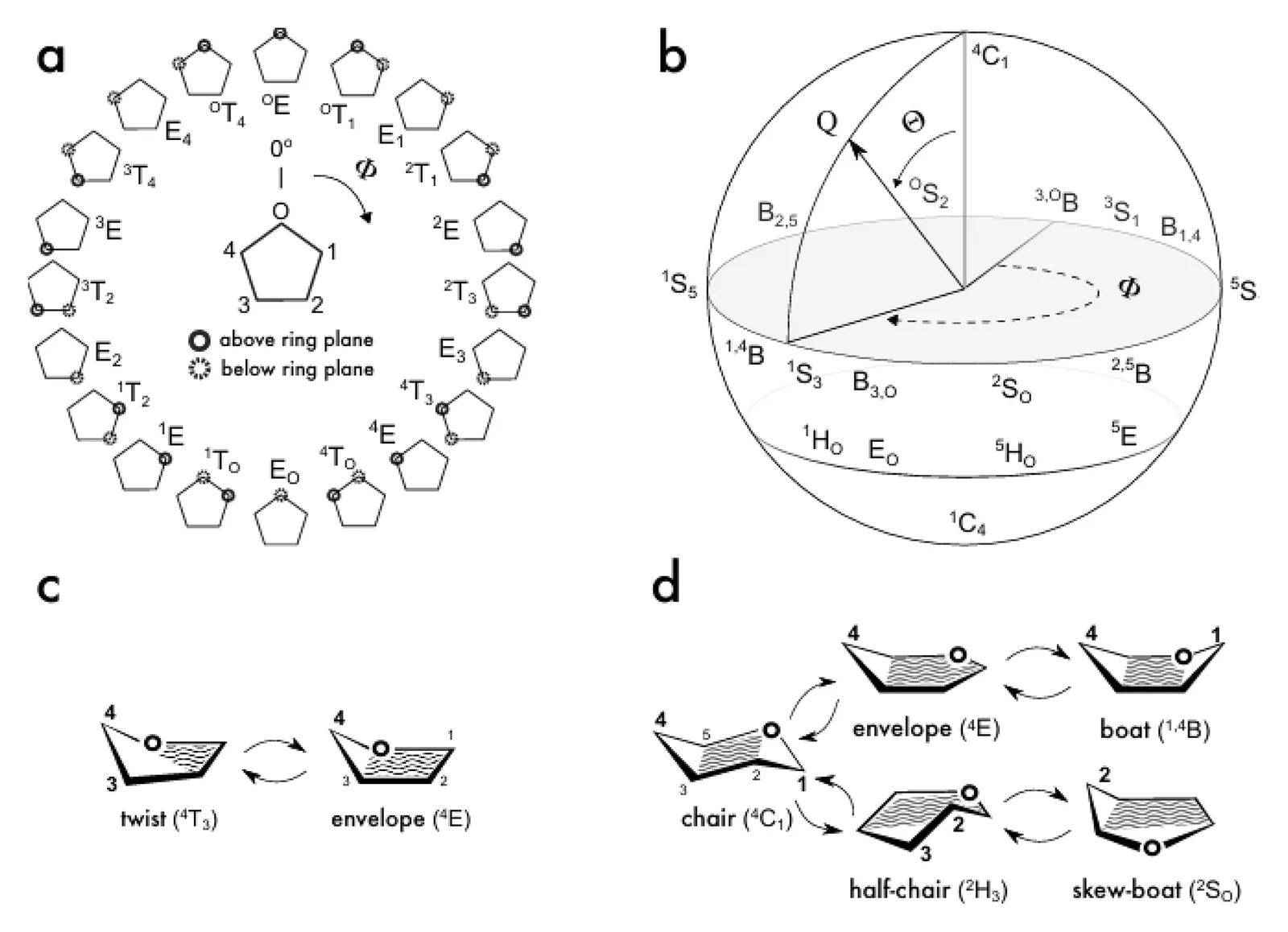
Conformational landscape of furanose and pyranose rings. A) Schematic representation of furanose ring puckering shapes, represented by a series of different envelopes and twists. B) The Cremer-Pople Sphere, representing the mathematical mapping of six-membered pyranose puckering coordinates (*Q, θ, ϕ*) onto a spherical surface. The low-energy ^4^C_1_ and ^1^C_4_ chairs sit at the North Pole (*θ* = 0˚) and South Pole (*θ* = 180˚) respectively. Along the equatorial plane (*θ* = 90˚), there is flexible pseudorotation between the boat (B) and skew-boats (S). Ring inversion from the North to the South pole requires navigating through high-energy half chair (H) or envelope (E) transition states. C) Structures indicating the planes and atom labels for furanose ring conformations. D) Structures indicating the planes and atom labels for pyranose ring conformations. Figure adapted from (Agirre 2017). Figure 3 alt text: Four-panel diagram illustrating carbohydrate ring conformations, featuring furanose puckering shapes, the Cremer-Pople sphere mapping pyranose coordinates, and chemical structures with standard atom labeling for both ring types.

To assess whether a modelled sugar ring is in the expected low-energy conformation, *Privateer* relies on the Cremer-Pople puckering coordinates (see Figure 3) (Cremer and Pople 1975). In 1975, Cremer and Pople introduced a general definition of ring puckering coordinates, providing a quantitative method for describing the puckering of non-planar ring systems. For both pyranose and furanose rings, the Cremer-Pople parameters can distinguish between different ring conformations, whilst quantifying the degree of distortion from idealised geometries.

For pyranose rings, the puckering coordinates are mapped onto a three-dimensional spherical coordinate system defined by three parameters: total puckering amplitude, *Q*, which quantifies the magnitude of displacement from planarity, and the angular coordinates *θ* and *ϕ*, which specify the directional orientation of the puckering (seeFigure 3). On this conformational sphere, the most thermodynamically stable, lowest-energy conformations occupy the poles. The ^4^C_1_ and ^1^C_4_ chair conformations reside at the North (theta = 0˚) and South (*θ* = 180˚) poles, respectively, characterised by opposite ends of the ring puckering in opposing directions. Conversely, the equator (*θ* = 90˚) represents highly strained, high-energy intermediates, such as boat (B) and skew-boat (S) conformations, where the ring ends are displaced in the same direction. Because transitioning between the stable poles and the strained equator requires a significant energy input larger than 15 kcal/mol, pyranose rings favour the stable ^4^C_1_ chair (Biarnés et al. 2007). Consequently, sugars that are modelled as high-energy conformations are typically the result of modelling errors, unless stabilised by an external factor, such as the catalytic environment of an enzyme active site.

Comparatively, furanose rings occupy a smaller conformational space. Puckering parameters are calculated using a similar framework, where the pseudo-rotation of the ring is along a flat two-dimensional plane rather than a sphere (see Figure 3). Unlike pyranoses, the conformational landscape consists of ten distinct envelope (E) conformations (where four atoms are coplanar and one is out-of-plane) and ten distinct twist (T) conformations (where two adjacent atoms are displaced on opposite sides of the plane).

**Figure 4:**
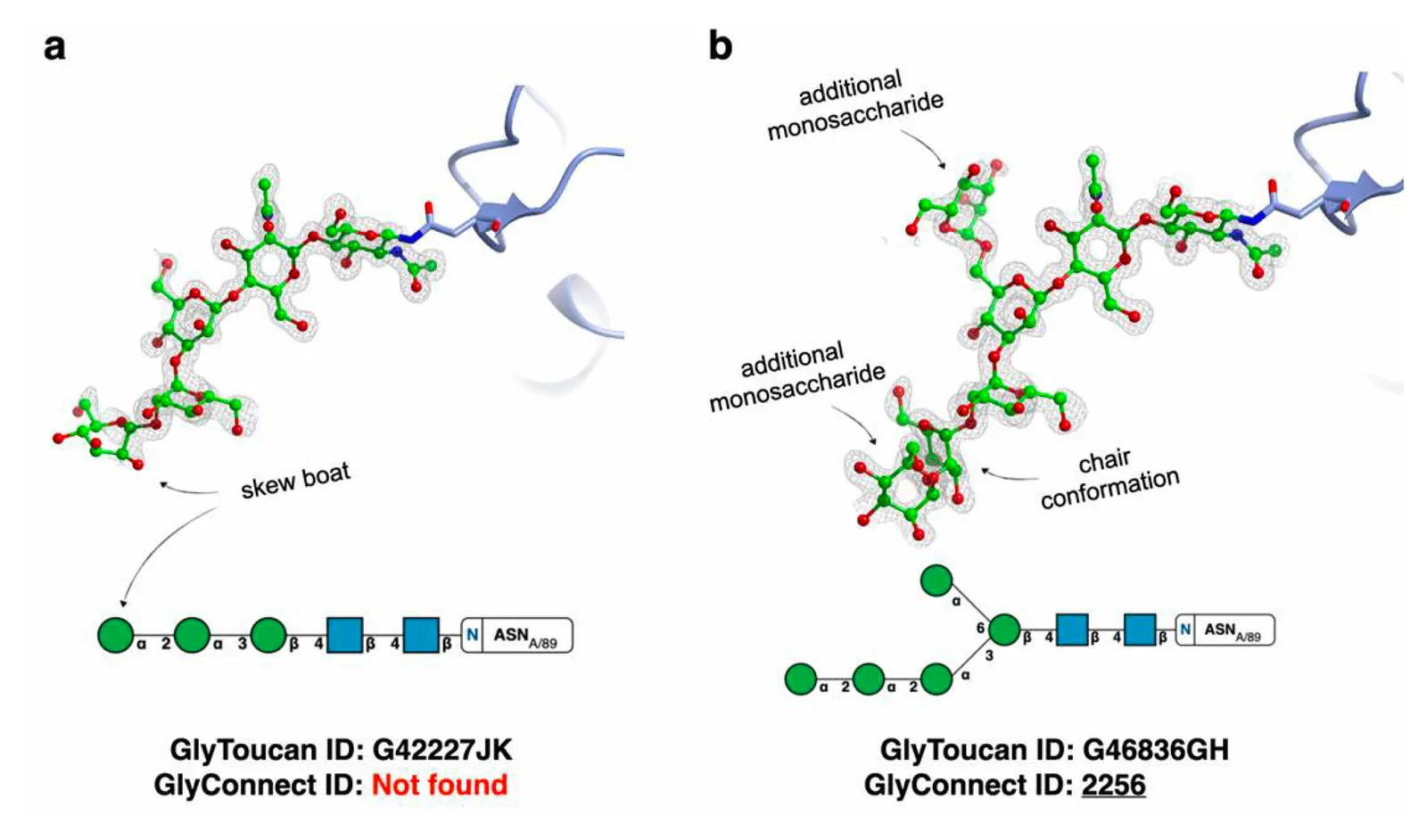
Fixing and extending glycan structures with *Privateer*. Analysis of 3QVP (Kommoju et al. 2011), an otherwise excellent model, using *Privateer* indicates a high energy skew-boat conformation, poor fit to density (RSCC of 0.41) and lack of *GlyConnect ID* for the *N*-glycan where it is found. Upon removal of a mismodelled water molecule and refinement of the *N*-glycan chain in *Coot*, the previously high-energy conformation turned into a ^4^C_1_ chair. Further inspection of the new maps revealed additional monosaccharides downstream, allowing the glycan chain to be extended. Figure 4 alt text: Two-panel figure showing a PDB-deposited glycan structure compared to the same structure once rebuilt according to Privateer’s validation output. The deposited structure shows a high-energy skew-boat conformation, which does not have a GlyConnect ID, whilst the re-built structure has all low-energy ^4^C_1_ chair conformation, and an extended glycan chain that has a GlyConnect ID.

In order to calculate the ring conformation of a modelled sugar, *Privateer* is able to identify carbohydrate chains within a structure and references them against a manually curated internal database, which was created by analysing the idealised, low-energy conformers’ coordinates in the PDB’s Chemical Component Dictionary (CCD) (Westbrook et al. 2015). For every supported monosaccharide, a set of ideal, energy-minimised Cremer-Pople coordinates are stored for comparison. *Privateer* extracts the three-dimensional coordinates of the ring atoms and calculates their Cremer-Pople parameters. By comparing the calculated coordinates (*Q, θ, ϕ*), the ring conformation is detected. Following this, *Privateer* calculates the RSCC for each sugar to measure its fit to the electron density. A sugar with an RSCC below 0.7 is flagged as a potential density-fit outlier because it is poorly supported by the local density. This provides an important complement to conformational validation, as an unusual ring conformation may represent either a modelling error or a genuine distortion supported by the density. A key example of this is for iduronic acid, where the ^1^C_4_ diagnostic check does not fully reflect the complexity of the glycan, which exists in a dynamic equilibrium between multiple ring conformations. In this case, high-energy ring conformations may be flagged by *Privateer*, but the calculated RSCC score can be used to identify whether the conformation fits the density. An example of a high energy ring conformation modelled in the PDB can be seen in Figure 4, showing a mannose pyranoside as a high-energy skew-boat conformation. *Privateer* flags this as a conformational outlier, and calculates an RSCC of 0.41, indicating to the user that this high-energy conformation is not supported by the local density.

### Updated restraints prevent the modelling of distorted sugars

Whilst *Privateer* is able to flag these high-energy conformations after structure refinement, it is crucial that refinement programs use correct geometry to avoid the misrefinement of sugar rings. In macromolecular structure solution, refinement programs typically rely on restraint dictionaries that contain prior chemical knowledge, including information about bond lengths, angles, torsions, chiralities and planarities, to interpret the chemistry of compounds in macromolecular structures. These are typically stored in Crystallographic Information Files (CIF) files within libraries like the CCP4 Monomer Library *(Agirre et al. 2023)*. However, these past carbohydrate restraint dictionaries were outdated, containing incorrect geometries (Atanasova et al. 2022). These dictionaries also frequently lacked explicit ring puckering restraints, resulting in refined structures which had skewed, high-energy ring conformations that fit noisy density rather than chemical reality (Atanasova et al. 2022).

To solve this systemic issue, new restraint dictionaries were regenerated for pyranose rings within the CCP4 suite, which were shown to help address conformational issues with a reduction in the gap between R_work_ and R_free_ (Atanasova et al. 2022). *Privateer* provided a list of the most frequently modelled sugar rings in the PDB, which were fed into the AceDRG restraint generation software in the form of CIF files. AceDRG sampled a vast ensemble of potential conformers (Long et al. 2017; Atanasova et al. 2022); the lowest-energy conformers were fed into *RDKIT* and *REFMAC5* to optimise the coordinates, and output a revised CIF dictionary (Murshudov et al. 2011; RDKit: Open-source cheminformatics. https://www.rdkit.org). Target values for bond length, angles, and torsions were updated, and outputted as a CIF restraint dictionary stored in the CCP4 Monomer Library. These restraint dictionaries and their generated conformers were then passed to *Privateer* for geometric validation. A key implementation resulting from this was the introduction of unimodal torsion restraints, which reflected the ideal, lowest energy ring conformation and, due to their unique naming, could be restrained separately from the rest of the molecule (Atanasova et al. 2022). These explicit, tight constraints were shown to effectively prevent refinement programs from driving sugar rings into high-energy conformations, ensuring they remained in their most chemically feasible states (Atanasova et al. 2022). It is important to note that these torsion restraints should only be activated in specific cases, where the user is certain the refinement program is mistakenly skewing the sugar due to poor density. For most cases, refinement should be performed without these restraints, as skewed sugars will most often be a sign of a modelling error elsewhere in the polysaccharide or its environment.

In addition to sugar ring conformational preferences, glycosidic bonds also have distinct conformational preferences. Just as the Ramachandran plot validates peptide backbone torsions, *Privateer* provides a similar tool for glycan chains (including *N-, O-* and *C-* glycans) by calculating torsion linkage preferences based on a validated set of glycosylated structures from the PDB (Ramachandran et al. 1963; Dialpuri et al. 2023). Branched glycan chains (including *N-* and *O-*glycans) contain a variety of highly flexible glycosidic bonds defined by specific torsion angles: *ϕ*, and *ψ* which are variable, and *ω* which is typically 180˚ (Dialpuri et al. 2023). The torsion angle of the linkage between a sugar and an amino acid residue is also of interest. To establish a reliable reference baseline, the PDB was mined for well-fitting (RSCC > 0.80) sugar rings in a ^4^C_1_ chair conformation (Dialpuri et al. 2023). By filtering for only these validated structures, a database of validated glycosidic and asparagine-sugar linkages was compiled, resulting in relatively clean clusters of preferred torsion angles that serve as a benchmark for each link. For a sugar linkage, *Privateer* calculates a Z-score which allows users to compare their modelled linkage torsion to the validated observations.

### *Privateer* ensures the modelling of biologically feasible glycans

Along with identifying issues regarding sugar chemistry, *Privateer* is also able to detect issues with their biology through the analysis of glycan composition. Due to both advancements in mass spectrometry and the elucidation of underlying biosynthetic pathways, glycomics databases contain an extensive repository of biologically feasible, naturally occurring glycans (Tiemeyer et al. 2017; Alocci et al. 2019; Bagdonas et al. 2020). *Privateer* leverages these data by extracting the atomic coordinates of a modelled glycan chain and translating its topology into the standardised WURCS (Web 3.0 Unique Representation of Carbohydrate Structures) notation (Matsubara et al. 2017; Bagdonas et al. 2020). By generating standard WURCS strings, *Privateer* significantly enhances the FAIRness (Findability, Accessibility, Interoperability and Reusability) of glycan structural data, bridging 3D macromolecular models with the broader glycoinformatics landscape. The WURCS code is cross-referenced against two key resources: *GlyTouCan*, an international glycan structure repository, and *GlyConnect*, a glycoproteomics databank (see Figure 2)(Tiemeyer et al. 2017; Alocci et al. 2019). This integration allows researchers to move beyond basic structural validation and enrich macromolecular models with contextual biological metadata, such as tissue specificity, cell types, and species of origin. *GlyTouCan* can also act as a bridge between PDB and *GlyConnect*; if a *GlyConnect* ID is not identified, it signals that this specific glycan topology has never been deposited – almost invariably revealing a modelling error (see Figure 4) (Tiemeyer et al. 2017; Alocci et al. 2019; Bagdonas et al. 2020). Furthermore, it was identified that some *N*-glycan structures showed incorrect α-linkages between *N-acetyl-d-glucosamine* and asparagine residues, resulting in sugars being built as ^1^C_4_ chairs, as opposed to the more favourable ^4^C_1_ chair for d-pyranoses (Agirre, Davies, et al. 2015). These unlikely links can be detected via cross-referencing against both *GlyToucan* and *Glyconnect databases* (Tiemeyer et al. 2017; Alocci et al. 2019; Bagdonas et al. 2020). Furthermore, establishing this interoperability provides a vital interface with functional glycomics workflows, such as connecting to *GlyGen* for glycan microarray binding profiles and *GlyConnect* for site-specific glycoproteomics (York et al. 2020). Cross-referencing against these functional and structural repositories ensures that modelled glycans are not only compliant with known biosynthetic pathways, but are also biologically meaningful and experimentally plausible (Bagdonas et al. 2020).

Another identified issue in carbohydrate-containing structures was the mismodelling of sugar anomers, leading to unfeasible or incorrectly modelled glycans (Lütteke 2009; Joosten and Lütteke 2017). Sugar anomers are a result of linear sugar cyclisation, upon which the carbonyl carbon (typically labelled ‘C1’) becomes a new chiral centre. This generates two distinct stereoisomers, also known as anomers: α and β. In the PDB, different anomers of the same sugar represent distinct CCD entries, such as beta-d-mannose (BMA) named alpha-d-mannose (MAN). This detail increased the likelihood of erroneously modelled anomers, where the α-anomer has been modelled where a β-anomer should be (Lütteke 2009; Joosten and Lütteke 2017). In order to address this issue, *Privateer* checks the detected sugar’s anomer against the three letter CCD code, to ensure the modelled anomer matches the CCD residue name. This validation allows *Privateer* to correct mislabelled or mismodelled sugar anomers, but also helps identify biologically feasible links, as selection of the wrong sugar anomer has resulted in erroneous links in some structures (Joosten and Lütteke 2017).

### The exception to the rule: validating *C-*glycans

Whilst tryptophan *C*-mannosylation (Trp-Man) is a relatively rare type of glycosylation, analysis of the PDB revealed structures containing this modification are frequently mismodelled, or entirely omitted (Minakata et al. 2021; Holland et al. 2025). This is likely a result of *C*-glycans favourably adopting the more uncommon ^1^C_4_ inverse chair conformation, rather than the more common ^4^C_1_ chair conformation adopted by most pyranoses in *N-* and *O-*glycans (Holland et al. 2025). This conformational flip minimises steric clashes between the hydroxymethyl group of the mannose and the indole ring of the tryptophan residue (Holland et al. 2025). When the incorrect, default ^4^C_1_ chair conformation is initially modelled, subsequent refinement can distort the sugar ring into high-energy conformations to force a fit to the density, often arriving at conformations other than the expected ^1^C_4_ inverted chair at low resolution, due to weak gradients in the electron density (Holland et al. 2025). To resolve this, validation of Trp-Man linkages has been added to *Privateer*, where the sugar linkage is checked to be alpha, and the conformation is checked as ^1^C_4_. The dictionary for the Trp-Man connection in the CCP4 Monomer Library has also been updated to contain unimodal torsion restraints, which need to be activated in order to reliably flip the ^4^C_1_ starting conformation into the correct ^1^C_4_ inverted chair (Holland et al. 2025).

### Implementation and impact

**Figure 5:**
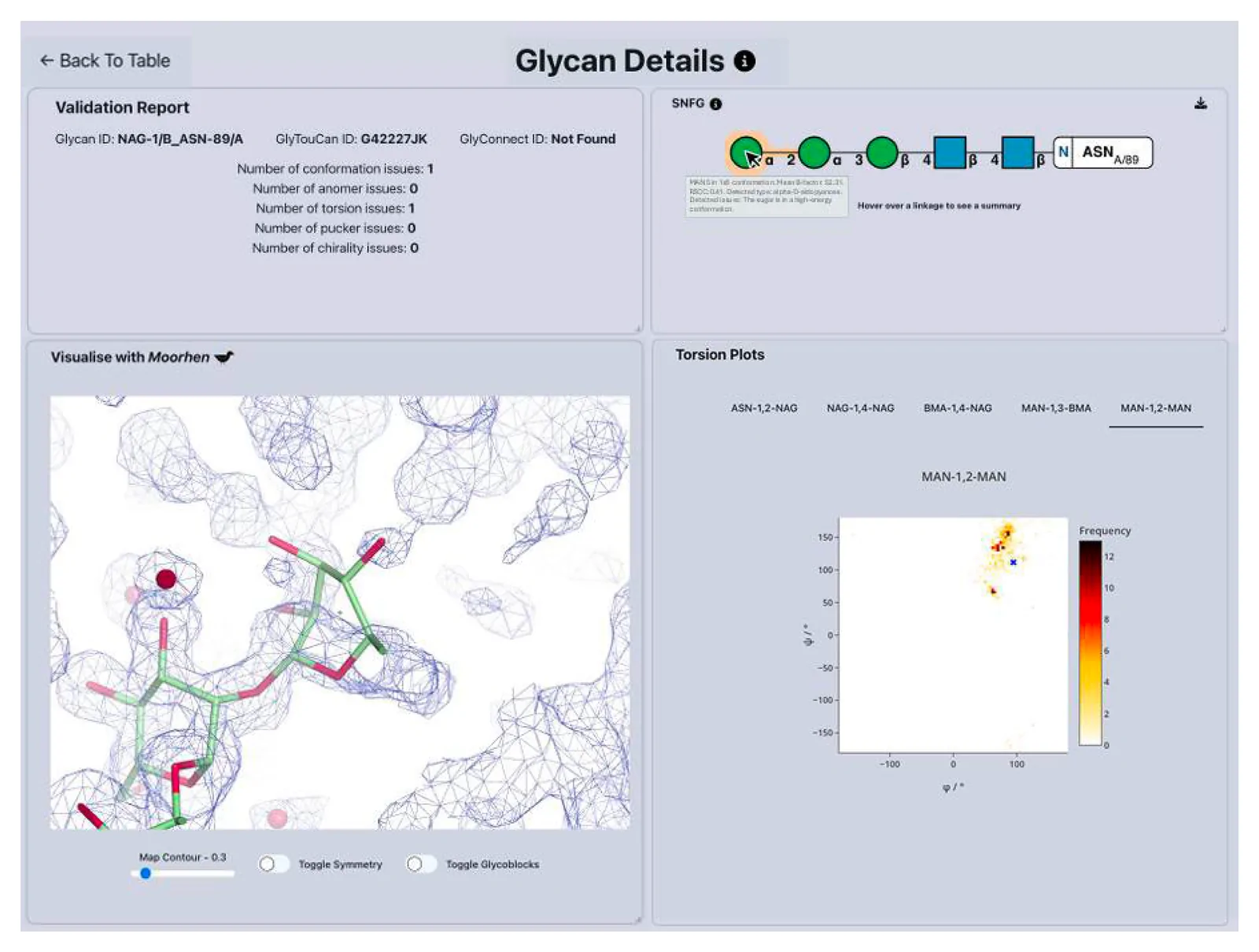
*Privateer* validation outputs for PDB ID 3QVP available via the *Privateer web application* (https://privateer.york.ac.uk/). (Top left) *Privateer* validation of PDB ID 3QVP (Kommoju et al. 2011). The results include cross-references to *GlyToucan* and *GlyConnect* databases (Tiemeyer et al. 2017; Alocci et al. 2019). Conformational, anomeric, torsion, puckering and chirality issues are also reported. (Top right) Symbol nomenclature for glycan (SNFG) diagram is drawn for the modelled glycan chain. Hovering over each sugar gives detailed information about the residue number, conformation, mean B-factor, RSCC, detected sugar type, and any detected issues by *Privateer*. Orange highlights over sugars and linkages on the SNFG indicated a detected outlier by *Privateer*. (Bottom left) An interactive panel for *Moorhen*, a *Coot*-based tool commonly used for model building and molecular graphics, which displays the model and density if supplied. Clicking on different sugars in the SNFG will navigate to that sugar in the *Moorhen* window. (Bottom right) Linkage torsion plots based on a validated set of structures from the PDB. Calculated linkage torsions from the sugar under investigation are plotted on the graph in blue. This results page is available for all glycan chains or ligands in a structure. Figure 5 alt text: Four-panel screenshot of the *Privateer* web application interface, showing glycan validation results, an interactive SNFG diagram, a Moorhen 3D molecular viewer, and linkage torsion plots.

*Privateer* has the ability to assess the chemical and crystallographic validation of carbohydrates and displays these results in an accessible form to the user (see Figure 5). *Privateer* presents its results in a series of tables and diagrams, translating complex validation into clear visual flags (see Figure 5). The validation results are clearly depicted onto standardised Symbol Nomenclature For Glycans (SNFG) diagrams, where orange highlights indicate a validation issue, flagging to the user to inspect this sugar (Varki et al. 2015). This allows model builders to instantly pinpoint sugar outliers, whether it be due to chemical feasibility or poor density fit. Further, the *Privateer* web app provides access to the *Privateer Database*, which enables researchers to analyse carbohydrate structures by providing pre-calculated validation reports for any PDB entry (Dialpuri, Bagdonas, Schofield, Pham, Holland, and Agirre 2024; Dialpuri, Bagdonas, Schofield, Pham, Holland, Bond, et al. 2024). Users can also query the entire repository of carbohydrate-containing PDB structures based on glycosylation type or linkage type (Dialpuri, Bagdonas, Schofield, Pham, Holland, and Agirre 2024; Dialpuri, Bagdonas, Schofield, Pham, Holland, Bond, et al. 2024). This allows glycobiologists interested in protein structures to easily access all deposited carbohydrate-containing structures.

Building on current integrations, several key developments will expand *Privateer’s* scope across model-building, cryo-EM, open-chain sugar support, and machine-learning predictions. Deeper integration into interactive model-building interfaces such as *Moorhen* will directly link residue-level outliers to local visual inspection and real-time correction, streamlining the iterative cycle between model building, refinement and validation within a unified interface. Furthermore, *Privateer*’s integration into highly accessible applications, like the *Privateer* web app and *Moorhen*, ensures that inspecting and validating glycoprotein structures is easier than ever, requiring nothing more than a web browser. Beyond classic pyranose and furanose rings, *Privateer* will extend its validation to open-chain monosaccharides, where the traditional Cremer-Pople ring puckering parameters are not applicable. Unlike X-ray crystallography, which requires rigid, highly ordered packing of macromolecules into crystal lattices, cryo-EM captures proteins in a near-native state (Atanasova et al. 2020). Because cryo-EM does not require this symmetry, it is a powerful approach for visualising highly flexible, heterogenous carbohydrate-containing structures. By embedding *Privateer* into widely used suites such as CCP4 and CCP-EM, carbohydrate validation is now a seamless part of the macromolecular structure solution pipeline for both X-ray and cryo-EM structures (Agirre et al. 2023; Burnley et al. 2023). This integration allows users to model, refine and validate sugars in a single workflow, using *Privateer*’s validation to guide their next step (see Figure 7). Future additions will incorporate Q-score analysis to assess density fit, analogous to RSCC calculation for X-ray crystallography structures (Pintilie et al. 2020; Burnley et al. 2023; Meng et al. 2023). Finally, a key role of *Privateer* going forward is for it to serve as a vital tool for evaluating AI-predicted models. Although recent frameworks such as *AlphaFold3* and *RoseTTAFold All-Atom* extend prediction to glycans, all models trained on historic PDB data risk propagating pre-existing errors (Abramson et al. 2024; Krishna et al. 2024). *Privateer* provides an essential independent benchmark, ensuring that AI-generated glycan models adhere to fundamental carbohydrate chemistry.

**Figure 6:**
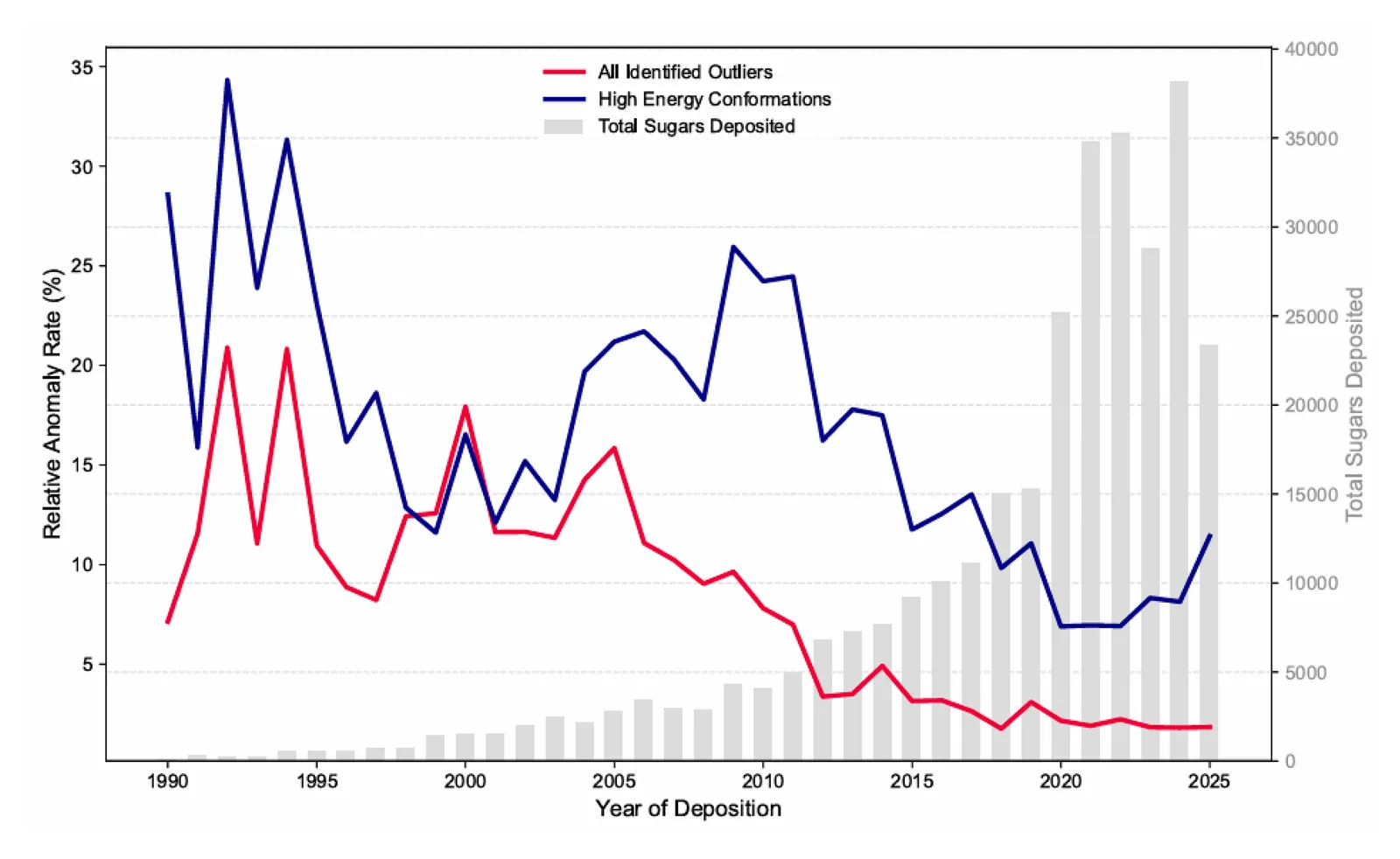
Annual trends of carbohydrate anomalies detected in the PDB. Grey bars (right-axis) represent the total number of sugar residues deposited per year. Lines track the relative percentage of annual sugar residues flagged as outliers by *Privateer* (left axis). Red lines denote any high-energy ring conformation by *Privateer*, while navy lines illustrate conformational outliers deviating from a chair conformation. The apparent decline in deposited structures for 2025 likely reflects structures that are still undergoing deposition review or on hold awaiting publication. Data was extracted from the *Privateer Database* (Dialpuri, Bagdonas, Schofield, Pham, Holland, and Agirre 2024). Figure 6 alt text: Dual-axis graph showing historical trends of annual carbohydrate depositions alongside the relative percentages of ring conformation and chair-deviation anomalies over time.

**Figure 7:**
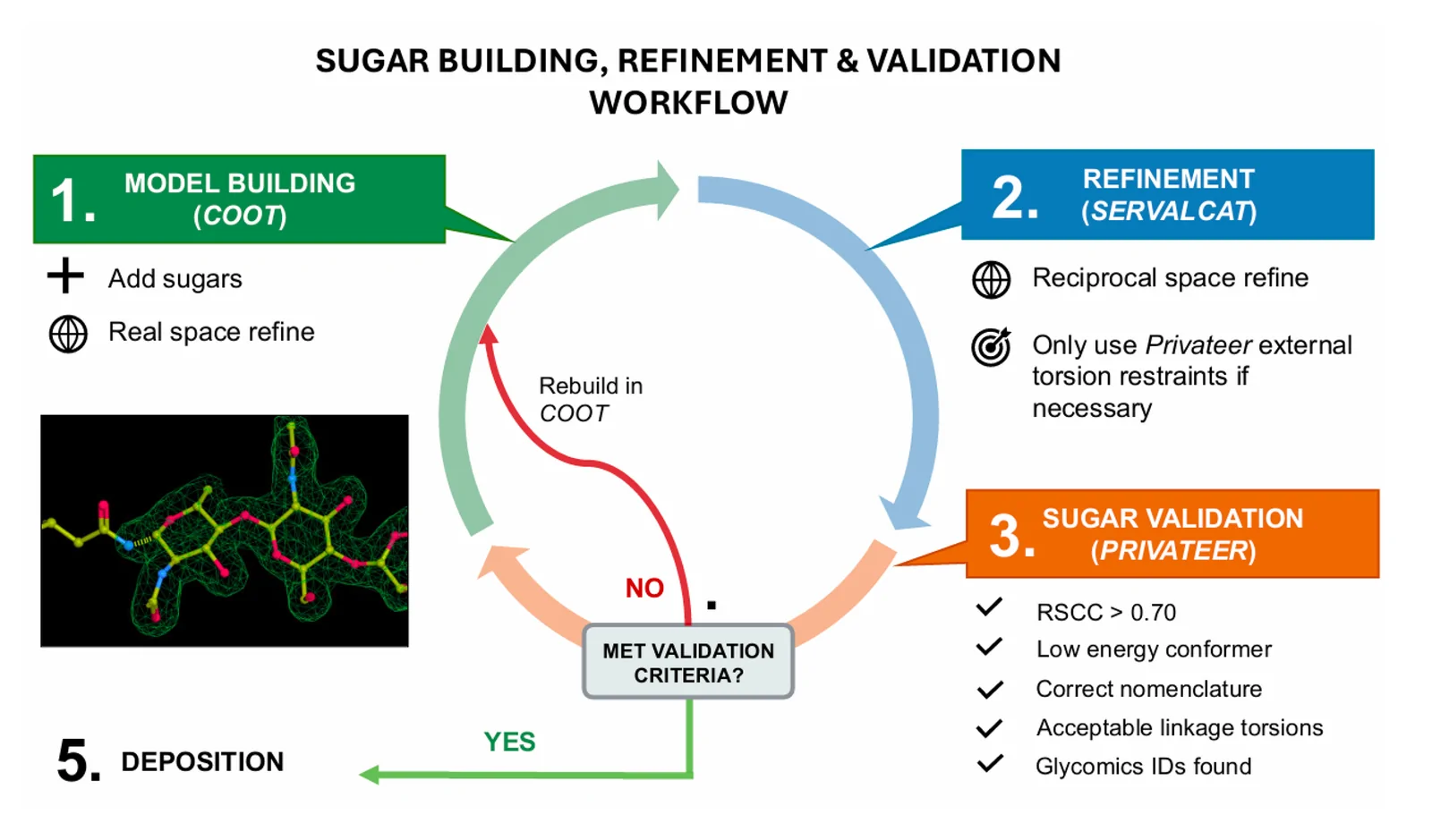
The role of *Privateer* as a validation tool for carbohydrate-containing structures. The generation of valid sugar-containing protein structures requires the iterative cycling of protein model building, where the atomic model is built into the density; refinement, in which the atomic coordinates are improved by balancing fit to electron density and geometric restraints; and validation using *Privateer*. Model building using *Coot* is the first step, followed by refinement using refinement engines such as *Servalcat (Emsley et al. 2010; Debreczeni and Emsley 2012; Yamashita et al. 2021; Yamashita et al. 2023)*. Structure refinement can be performed using external restraints generated by *Privateer*, but these should be used in specific use-cases, not as default. Validation is performed by *Privateer*, highlighting issues with the sugar chemistry or fit-to-density. The user can continue the loop if *Privateer* has flagged any issues. Figure 7 alt text: Circular flowchart showing the iterative cycle of carbohydrate model building with *Coot*, refinement with *Servalcat*, validation with *Privateer*, and final database deposition.

Historically, carbohydrate modelling has been prone to errors, including distorted ring conformations, mislabelled anomers, unexpected linkages, and poor density fit (Lütteke 2009; Agirre, Davies, et al. 2015; Agirre et al. 2017; Joosten and Lütteke 2017). Today, *Privateer* has streamlined this process, making it straightforward to generate chemically accurate, well-modelled, carbohydrate-containing structures. Analysis of incorrectly modelled sugars (as detected by *Privateer*) showed that the number of outliers has reduced from ~ 20% to less than 5% (see Figure 6). More specifically, the number of high energy conformations has reduced from ~ 30% in the 1990s to less than 10% today. Over the past decade, *Privateer* has evolved from a geometric validation tool into a key multi-functional validation tool for carbohydrates in structural biology. By tackling the underlying complex chemistry, combined with validation of density fit, the software has elevated the baseline quality and reliability of glycoprotein structures deposited in the PDB. *Privateer’s* contribution to the improvement of carbohydrate validation methods has allowed structural biologists who may not be experts in sugar chemistry or glycobiology to build complete, biochemically feasible carbohydrate structures. These structures further the understanding of glycobiology as a whole. Ultimately, by lowering the barrier to entry, *Privateer* ensures that high-quality carbohydrate models are no longer the exception in structural biology, but the standard.

## Data Availability

The *Privateer* source code can be found at https://github.com/glycojones/privateer.
